# Acid—Muddy-Like

**Published:** 1883

**Authors:** 


### PART SECOND.

### EXPECTORATION.

---

**ACID** (sour): Ambr., Ant. t., Ars., *Bell.*, Bry., CALC., Carbo an., Carbo v., Cham., *China*, Con., Graph., Hepar, Ign., Ipec., *Kali c.*, Lyc., Mag. m., Merc., Natr. c., Natr. m., Nitr. ac., NUX V., Petr., PHOS., Ph. ac., Plumb., *Puls.*, Rhus, Sabina, Sepia, Stann., *Sulph.*, Tarax.—4.

**ACRID**: *Alum.*, Amm. c., *Amm. m.*, Anac., *Ars.*, Carbo v., *Caust.*, Cham., Con., Ferr., Ign., Iod., Kreos., Lach., Lyc., Mag. m., *Merc.*, Mezer., Natr. m., Nitr. ac., Nux v., Phos., *Puls.*, Sepia, *Sil.*, Spig., Squil., Sulph., Sul. ac.—4.

**AFTERNOON**: Chin. s., Coc. c., Phos.

— in open **air**: Chin. s.

— **bloody**: Alum., Lyc.

— — streaked phlegm: Alum.[6]

— **difficult**: China.

— **gray** yellow sputa: Anac.[6]

— **mucous**: Amm., *Bad.*

— **slimy**: Chin. s.

— — 5.30 P. M.: Hydras.

— **tenacious**: Anac., Ars.

— **thick**: Eucalyptus.

— **viscid**: Naja.

— yellow: Anac.

— — 12–3 P. M.: Calc. s.

**AIR**, expectoration on getting into cold: Plan.

— — **amel.** in cold or open air: Calc. s.

**AIR**, expectoration on going into cold air: Plan.

— **open** agg., during day: Nux v.

— — when in: Sac. alb., Sepia.

— — — mucous expectoration: Kobalt.

— — afternoon: Chin. s.

— — walking in open air: Nux v., *Sac. alb.*

— — bloody expectoration when walking in open air: Merc.[6]

**AIR PASSAGES**, seems to come from: Arn.

**ALBUMINOUS**: Amm. c., Amm. m.,[4] Arn., *Ars.*, Asaf.,[12] *Bar.*, Bor., Bov., *China*, Cocc. c., *Ferr.*, Kali b., *Laur.*, *Mezer.*, Petr., *Senega*, Sil., Sulph., *Stann.*—5.

— night in bed, after cough: Sulph.

**ALMONDS**, tasting like: Caust., Digit.—2.

**ASH** colored clots: Arundo.

**BALLS**, in shape of: *Arg. n.*, *Coc. c.*, Lyc., *Sil.*, STANN., Sulph.

**BATH**, expectoration after a bath, in morning: Calc. s.[1]

— — of blood after a sea bath: *Mag. m.*[5]

**BED**, expectoration evening in: Calc.

— — on getting into bed, before midnight: Sepia.

— — morning in: *Calc.*

— — — on rising from, coughs up blood: Ferr.

— — on sitting up in: *Phos.*

**BILIOUS** (like bile): Baryta, Digit., Puls., Samb.[7]—5.
— in taste: Puls.[1]
**BITTER**: Acon., Ailan.,[7] Arn., *Ars.*, Bry., Calc., Canth.,[11] CHAM., China, *Cist.*,[1] Coloc., Con., *Dros.*, Ign., Kali c., *Lyc.*, *Merc.*, Merc. sol.,[1] Natr. c., Natr. m., *Nitr. ac.*, *Nux v.*, Pau. p.,[1] PULS., Sabad., *Sepia*, Stann., Sulph., *Verat.*—5.
— in morning: Ailan.[7]
**BLACKISH**: Arn., Bell., *China*, Elaps, *Kali b.*, Lyc., *Nux v.*, Ox. ac., Puls.,[1] Rhus.—1 and 5.
— day and night: Lyc.
— with blackish grains: China.[12]
— lumps (dots) in centre: Arn., Ox. acid.—5.
— yellow: Hydro. ac.
**BLOODY** (spitting of blood, etc.): ACON., Ailan.,[7] All. sat., Aloe, Alum., Ambr., Am. bro., *Amm. c.*, Amm. m., Anac., Ant. cr., Ant. s., Arg. n.,[11] ARN., *Ars.*, Arum m.,[6] Asar., Aur., Bad., *Bell.*, Bism., Bond., *Bor.*, Both., *Bry.*, *Cact. gr.*, *Calc.*, Calc. caus.,[6] *Canth.*, Caps., Carbn. ox., Carbo an., *Carbo v.*, Cham., *China*, Cina, Cist., *Con.*, Cop., *Croc.*, Crot. h., Cupr., Daph., Der., Diad., *Digit.*, Dios., *Dros.*, *Dulc.*, Elaps, Erigeron,[7] Eugen., Euphr.,[2] FERR., Fluor. ac., Gam., Gels., Graph., Guai.,[7] *Ham.*, Hell., Hepar, Hydro. ac., *Hyos.*, Indigo, Iod., IPEC., Jug. c., Kali b.,[10] Kali c., Kali iod., Kali hypermang., Kobalt., Kreos., Lach., Lachn., *Laur.*, LED., *Lyc.*, *Mag. c.*, Mag. m., Mang., *Merc.*, Merc. c., *Mezer.*, *Millef.*, Mur. ac., Natr. c., *Natr. m.*, *Nitr. ac.*, *Nitrum*, Nux m., Nux v., Œna., *Op.*, Penth., PHOS., Ph. ac., *Plumb.*, Psor., PULS., *Rhus*, Ruta, Sabad., *Sabina*, Salic. ac., Sang., *Secale*,

**BLOODY**—(*Continued*).
Selen., *Sepia*, *Sil.*, Sol. m., Squil., Stann., Staph., SULPH., *Sul. ac.*, Tarax., Thuja, *Zinc.*—1 and 5.
— **acrid**: *Amm. c.*, *Ars.*, *Canth.*, Carbo v., Hepar, KALI C., *Nitrum*, Rhus, SIL., Sulph., Sul. ac., Zinc.—5.
— **afternoon**, in the: Alum., Lyc.
— — 1 P. M.: Clem.
— — 2 P. M.: Nux v.
— — 5 P. M.: Mag. c.
— **black**: Arn., Bism., Canth., China, Croc., Digit.,[12] Dros., ELAPS, Kali b.,[1] Nitr. ac., Nux v., Ph. ac.,[12] Puls., Zinc.[12]—5.
— **bright** red color: Acon.,[12] Amm. c., *Arn.*, Ars., BELL., Bry., *Calc.*, Canth., Carbo an., *Carbo v.*, China, Digit., Dros., DULC., Ferr., HYOS., *Ipec.*, Kobalt., *Led.*, *Merc.*, Millef.,[12] Nitrum,[12] Nux m., Nux v., *Phos.*, Puls., *Rhus*, Sabad., SABINA, *Secale*, Sepia, Sil., *Zinc.*—4 and 5.
— bright red and foaming: Led.[5]
— — pure blood, morning and during day: Zinc.[7]
— — — as from larynx: Kobalt.[5]
— **brown**: BRY., *Calc.*, CARBO V., Con., Puls., *Rhus*, Sil.[6]—5.
— **coagulated** (in clots, masses, etc.): Acon., *Arn.*, *Bell.*, Bry., Canth., Carbo an., Caust.,[24] CHAM., *China*, Collins., Con., *Croc.*, Dros., *Ferr.*, *Hyos.*, *Ipec.*, Kreos., Mag. m., *Merc.*, *Nitr. ac.*, Nitrum,[7] Nux v., Ph. ac., *Puls.*, RHUS, *Sabina*, Secale, Sepia., Stram.,[24] Stront.[24]—5.
— — brown: Bry.[12]
— — in lumps, enveloped in mucus: Collins.[5]
— — pure coagulated blood in evening: Sepia.[6]
— **chronic** expectoration of: Sul. ac.[5]

**BLOODY**, when **clearing** the throat: Amm. c.
— on **coughing**: Alcoh., Anac., Crot. h., Ferr., Ipec., Kali c., *Led.*, Nux m., Thea.
— — — evening: Natr. c.
— — — morning: Bell., Sepia.
— during whooping cough: Con., Guare.
— **dark**: Acon., *Amm. c.*, Amm. m.,[24] *Ant. cr.*, *Arn.*, *Asar.*, Bell., *Bism.*, *Bry.*, *Canth.*, Carbo v., CHAM., *China*, Con., CROC., *Cupr.*, Digit., Dros., *Elaps*,[10] Ferr., *Kreos.*, Led., *Lyc.*, *Mag. c.*, Mag. m., Merc., Mur. ac.,[7] Nitrum, *Nitr. ac.*, *Nux m.*, NUX v., Phos., *Ph. ac.*, *Puls.*, Secale, *Selen.*, *Sepia.*, *Sulph.*, Sul. ac.—5.
— **erection**, after a violent: Natr. m.
— **evening**, when coughing: Natr. c.
— — after lying down: Sepia.
— — Compare with Evening Expectoration, in general.
— from least **exertion**: Ipec.[13]
— **frothy** (foaming): *Arn.*, Dros., Ferr., *Led.*, Opium, *Phos.*, *Silicea.* —5.
— **hæmorrhage.** Compare with Bloody Expectoration.
— — after great loss of blood: Ars., *China*, Ferr.—6.
— — — expectoration of blood or pus after hæmorrhage from lungs: Plumb.[7]
— — after coffee: Nux v.
— — in drunkards: Ars., Hyos., *Nux v.*, Op.—6.
— — after severe exertion: Acon., *Arn.*, Ferr., Puls., Rhus.—6.
— — after suppression of hæmorrhoidal flow: Acon., *Carbo v.*, *Led.*, Lyc., *Nux v.*, Phos., *Sulph.* —6.

**BLOODY**, **hæmorrhage** in lying-in women: *Acon.*, *Arn.*, *China*, Hyos., Ipec., *Puls.*, Sulph., Trillium.—6.
— — after suppression of menses: *Acon.*, Ars., *Bell.*, *Bry.*, Con., *Ferr.*, Millef., Phos., PULS., Senec., Sepia, Sulph.—6.
— — with onanists: Carbo v., *China*, *Ferr.*, Nux v., Phos., Sulph.—6.
— — in phthisical patients: *Acon.*, *Arn.*, Ars., Cham., China, Dros., Ferr., Lach., *Millef.*, *Phos.*, Puls., Sepia, Stann., Sulph., Sul. ac. —6.
— — after wine: Acon. (Raue.)
— — after whisky: Merc., Puls. (Raue.)
— — hæmoptysis, tickling cough, tightness of chest, cannot lie down; mind calm: Ham.[7]
— — hæmoptysis after loss of blood; heat all over, especially with pain between scapulæ; in drunkards or from suppressed menses: Ars.[7]
— — hæmoptysis, preceded by a sensation in pit of stomach as if a hard body were there; after the cough, a fetid sweat breaks out, followed by weakness in head: Hepar.[6]
— — hæmoptysis and cough, after typhus: Sul. ac.[7]
— — frequent blood spitting, severe chest pains, afternoon fever and morning sweat: Kreos.[7]
— — periodical blood-spitting, with greenish, yellow, pus-like sputa: Kreos.[7]
— — raising of blood, followed by copious mucus expectoration, evening: Agnus c.[7]
— — spitting of dark, coagulated blood, pain in lower part of chest; anguish, shuddering; qualmishness: Puls.[7]

**BLOODY, hæmorrhage,** slight spitting of black, thick, viscid blood, or bright red, frothy blood mixed with mucus and coagula: An.[7]

— — spitting blood in small quantities, with pus-like phlegm and pain in right chest: Illicium.[7]

— — cough with expectoration mixed with drops of blood, preceded by a scraping sensation in chest: Staph.[6]

— — cough with raising of bloody mucus, early morning: Bell.[6]

— — cough with raising of black, coagulated blood from throat and nose: Nitr. ac.[6]

— — cough with raising of bloody phlegm, weight on chest, short breath especially when ascending: Amm. c.[6]

— — cough, raising sanguineous mucus, preceded by stitches in side: Zinc.[6]

— — continual irritation, with cough and spitting of blood, accompanied by torturing oppression of breathing: Arg. n.[6]

— — deep hollow cough with discharge of pure blood: Sil.[6]

— — discharge of blood either pure or mixed with mucus, with stitches in chest: Nux m.[6]

— — expectoration of blood, following an itching in throat: Amm. m.[7]

— — roughness and bloody taste in mouth, succeeded by cough and expectoration of bright red blood, with burning and weight in chest, heat and redness of the face, and trembling of whole body: Amm. c.[6]

— on **hawking**: Cham., Ferr., Hyper., Nitrum.—1.

— after an **itching** in throat: Amm. m.[7]

**BLOODY,** while **lying** down, morning: Merc. sol.

— after a **meal**: Sepia.

— during **menses**: Natr. m., *Phos.*

— — — and before, with dry cough, burning pain and soreness in chest, morning and evening: *Zinc.*

— — — first day of menses, with fatiguing hacking cough: Phos.

— **moon** during full: Nitrum.

— **morning**: Acon., Alum., Ferr.,[12] Indigo, Mezer., Nitr. ac.,[6] Selen.,[7] Sepia, Sil., Sol. t. æ.—1.

— — compare with Morning Expectoration in general.

— — in bed: Nitr. ac.

— — on coughing: Bell., Sepia.

— — while lying down: Merc. sol.

— — during menses: ZINC.

— — before rising: Nux v.

— — when walking: Zinc.

— at **night**: Arn., Ars., *Ferr.*, Mezer.,[1] Puls.,[6] Rhus, Sulph.[1]—12.

— at **noon**: Silicea.

— **pale**: Amm. c., Arn., Ars., Bell., Borax, *Bry.*, Calc., Canth., Carbo an., Carbo v., China, Digit., Dros., Dulc., Ferr., Graph.,[24] *Hyos.*, Ipec., Kreos., Laur., Led., Mag. m., Merc., Natr. c., Nitrum, Nux m., Phos., Puls., Rhus, Sabad., Sabina, Secale, Sepia, Sil., Sulph., Zinc.—5.

— in **points** and copious: LAUR.[5]

— **mixed** with pus: China.[5]

— **red**, see Bright Red.

— **respiration**, from violent effort at: *Secale.*

— before **rising**, morning: Nux v.

— after **sea bath**: *Mag. m.*

— **slimy** expectoration after hæmoptysis: Ant. t.[7]

— **streaked**: Acon., Alum., *Amm. c.*, Anac., Ant. cr., *Arn.*, *Ars.*, Bism., *Bor.*, BRY., Calc., *Caust.*,

**BLOODY, streaked.**—(*Continued*). *China*, Cina, Cocc., Con., Crotal.,[24] Cupr., Daph.,[11] Digit., Dros., Dulc., Eugen.,[24] Euphr., FERR., Hepar, Iod., *Ipec.*, Kali b., Kali c., Kreos., Lach.,[24] Lachn., *Laur.*, *Lyc.*, Mag. m., Merc., *Natr. m.*, Nitr. ac., Nux m., *Op.*, *Phos.*, Puls., *Sabina*, Secale, *Selen.*, Seneg.,[11] Sepia, Sil., Spong., Squil., Sul. ac., Tereb.,[7] *Zinc.*—5.

— — in afternoon: Alum.[6]

— while **suckling** child: *Ferr.*

— after **talking**: Hura.

— **thick**: Cupr.

— **thin**: Ferr., Nux m., Sabina.

— **throat**, when clearing the: Amm. c.

— — after itching in: Amm. m.

— after venesection: Amm. caus.

— **viscid**: CROC., *Cupr.*, Mag. c., Secale.—5.

— while **walking**: Cham., Merc. sol., Sul. ac., Zinc.

— — — morning: Zinc.

— while **working**: Merc. sol.

**BLUISH** expectoration: Arundo, *Kali b.*, Natr. ars., Sulph.

— gray: Coc. c.

— night in bed: Sulph.

**BREAKFAST**, expectoration after: Sepia.

**BRICKDUST** color: Bry., *Phos.*, Rhus.

**BRONCHI**, seems to come from: Chin. s., Gastein, Hydrocotyle, Upa.

— — — — — in morning: Ipec., Lyc.

**BROWNISH**: Arum it., *Carbo v.*,[5] Sil.—1.

— dirty-brownish sputum, not rusty: Caps.[7]

— morning: Agar.

— — 10 A. M., while sitting: Mag. c.

— tubercles: Phos.

— yellow: Lyc.

**BUBBLES** of air, agg. morning in open air: Kobalt.

**BURNED**, when dry on the floor looks as if: *Phos.*

**CABBAGE**, tasting like boiled: Sulph.[2]

**CATARRH**, has odor of an old: *Ign.*

— tasting like old: *Bell.*,[11] *Ign.*, Mezer., *Puls.*, Sabin., *Sulph.*, Zinc.—5.

**CHALK**, tasting like: Ign., Nux v.—5.

**CHEESE**, like: Bry., China, Fago, Lyc., Pimp., Puls., Salic. ac.

— tasting like: China, Lyc.—2.

— tasting like old: Thuja.[12]

— — — putrid: Kali c., Phos., Zinc.—5.

**CHEST**, **aching** in, amel. by expectoration: Asaf.

— expectoration from deep in lungs: Chelid.

— expectoration of small, transparent lumps, almost without cough, relieving the lungs: Agar.[7]

— **heat** in, amel. by expectoration: Cham.

— **mucus**, accumulation of (in general), in chest: Arg., Arg. n.,[1] Asar.,[1] Aur., Bar., Bell., Brom., *Calc.*, Canth., Cham., Cupr., Dulc., Euphr., Graph., Guare., Hepar, Hipp., Iod., Ipec., Lyc., Mosch.,[1] Natr. m., Nux v., Sac. alb., Senega, Sepia,[1] Staph., Sulph., *Zinc.*—12.

— — — — evening: Crot. t.[12]

— — — — morning: Caust., Natr. m., Nux v.[1]—12.

— — rattling of, amel. by expectoration: Sulph.

— **oppression** of, relieved by expectoration: Manc.[7]

— **pain** in, amel. by expectoration: Chelid., Euonymus.

**CHEST**, **pressure** in sides of, during expectoration: Angust.
— **shocks** in, from hawking: Raph.
— **soreness** in, from expectoration upward: Lyc., Zinc.
— — — from hawking: *Calc.*
— **tension** in, amel. by expectoration: Calc.
— **weak** feeling in, copious expectoration of thick yellow mucus with: Ruta.[7]
**CHOCOLATE** and milk, like: Hura.
**COMPACT**: Clematis.
**CONGEALED**, morning: Cereus s.
**CONSISTENT**: Dign., Lyc.
**CONSTANT**, almost, day and evening: Arg. m.[7]
**COOL** (cold): Bry., Calad.,[10] Cann. s., *Coral.*,[10] Lach.,[10] Merc., Nitr. ac., Nux v., *Phos.*, Rhus, Sac. alb.,[1] Sulph.—5.
— gray-greenish cool mucus: Rhus.
— mucus, tasting flat: Bry.[12]
**COPIOUS** (profuse, abundant): Agar.,[12] Alum., Amm. c., Ammoniac., Ant. cr., Ant. t., ARS., Asar., *Bism.*, Bry., Cact. gr., CALC., *Carbo v.*, Caust., *China*, *Cicuta*, Cina, Cocc. c., Cod., Copaiba, Cupr. n., Cyc., Daph., Dios., *Dros.*, *Dulc.*,[12] Euph., EUPHR., Eupat. per., *Ferr.*, Gastein, Graph., Guai., Hepar,[24] Hydras., *Iod.*, Kali b., *Kali c.*, Kali iod., Kobalt., Kreos.,[7] *Laur.*, Led., LYC., Merc-i-rub., Œna., Petro., *Phos.*, Ph. ac., PULS., Ruta, *Samb.*, Senega, SEPIA, *Sil.*, Squil., STANN., *Sulph.*, Sul. ac., *Thuja*, Trif. p., Verat.,[11] Wies.—1 and 5.
— bright-red bloody points: LAUR.[5]
— **day**: Cic.[6]
— **evening**, on lying down: Graph.
— **free** expectoration relieves cough: Ailan.[7]

**COPIOUS**, **morning**: Alum.,[6] Calc., Calc. s., Coc. c., Digit., Euph., Kobalt, Zing.[7]—1.
— — 9 A.M.: Kobalt.
— **mucous** expectoration: Alum., Cact. gr., Caust., Coc. c., Hydras., Iod., Puls., Ruta, Senega, Sepia, Squil.—5.
— — yellow: CACT. GR.
— **night** and morning: Carbo v.[7]
— while **vomiting**: Coc. c., Eupat. per.
**COPPER**, tasting like: Cupr., Kali c., Lach., Natr. m.—5.
**CORROSIVE**: Iodine.[5]
**CRUMBLY**: Ox ac.
**DARK**: Aqu. petr., Bism., Cupr., Kali b., Œna., Nux m.[7]—1.
— morning: Naja.
— and offensive: Ars.
**DAY**, expectoration in the, night without: Acon., Alum., Amm. c., Anac., Ang., Ant. t., Arg., Arn., ARS., Asaf., *Bell.*, Bry., *Calc.*, Caps., Carbo an., Caust., CHAM., China, Cocc., Colch., *Con.*, Euphr., *Graph.*, Guai., HEPAR, *Hyos.*, Kali c., Lach., Lyc.,[2] Mag. c., Mag. m., Mang., MERC., *Nitr. ac.*, *Nux v.*, Op., Petro., Phos., PULS., Rhus, *Sabad.*, Samb., SIL., Squil., Stann., *Stront.*, Sulph., Verat., Zinc. —5.
— **agg.** in daytime: Cicuta.
— and evening, yellow or gray, often cold mucus, generally of a sour or sweetish, sometimes of a bitter, putrid, or metallic taste, or finally of a clear dark red blood: Nux v.[2]
— a gray, greenish, sometimes purulent expectoration of an offensive, somewhat sour taste: Carbo an.[2]
— and morning. See under Morning.

**DAY** and night with copious, easy expectoration: Dulc.[2]
— pus, mixed with dark coagulated blood, or of tenacious mucus, having a flat, saltish, or sour taste; or, more rarely, a repulsive sweetish taste: China.[2]
— saltish mucus, or bright red blood mixed with coagula: Hyos.[2]
— scanty, tenacious, mucous expectoration of a bitter or offensive taste: Cham.[2]
— sputa of blood mixed with clots: Nitr. ac.[7]
— thin acrid, yellow-purulent mucus, often mixed with bright red somewhat clotted blood, and of a repulsive or saltish taste and offensive odor: Merc.[2]
— tenacious yellowish mucus, of a repulsive sweet taste; or else of bright red blood: Sabad.[2]
— yellow, tenacious mucus of a bitter or saltish, sometimes also a sour or putrid taste: Verat.[2]
— watery, but tenacious, yellowish, sometimes purulent expectoration of mucus mixed with clots of blood, of a fatty taste and offensive odor: Mag. m.[2]
— white, thickish, loose expectoration, looking like boiled starch, but not transparent, without taste or smell: Arg.[6]

**DENSE**: Kali b.

**DESIRE** for: Coca, Lyc., Senega.
— — afternoon: Ferr.

**DIFFICULT**: Ailan., *All. sat.*, Alum.,[7] Ambra, Ang., Aqu. petr., Arn., Ars., Arum tr., Arundo, Asc. t., Aspar., Atropia, Aur., Bar., Bor., Bov., Bry., Camph., Cann. s., Canth., Caust., Cham., *Chelid.*, China, Chlo., Cina, Coca, *Coc. c.*, Con., Cop., Coral.,[12] Crot. t., Cupr., Der., Digit., Dign., Dros., Euphr., Ferr., Hydrocotyle, Hyos., Ign., Iod., Jatro., Kali b., Kali c., Kreos., *Lach.*, Lyc., Mag. c., Mag. m., Mang.,[7] Natr. ars., Nitr. ac., Nux m., Nux v., Œna., Osm.,[12] Ox. ac., Par., Pau. p., *Phos.*, Plan., Plumb., Puls., Ratan.,[6] Sap., Seneg.,[11] Sepia, *Squil.*, Stann., Staph., Stram., Sulph., Sul. ac., Tarent., Vic., Zinc.—1 and 5.
— **adhering** to throat, causing choking: Kali b.[5]
— — — — teeth and lips: Kali b.[5]
— **afternoon**: China.
— at 2 A. M.: Chin. s.
— expectoration is difficult to raise after being loosened; must be swallowed: Ambr., Arn., Calad., Cann. s., *Caust.*, Coca,[1] Con., Dig., Dros., Eugen.,[24] Gels.[1] *Kali c.*, Lach., Mur. ac., Nux m., Osm., Sepia, Spong., Staph., Zinc.[1]—5.
— sputa followed by gaping: Op.[7]

**DREAMS** of expectoration of pus and blood: Hepar.

**DRINKING**, amel. after: Amm. caus.

**DRY**: Sin. n.

**DUNG**, tasting like: Calc., Carbo an., Cham., Merc.,[5] Sepia, Verat. —2.

**DUST**, as if mixed with: Phos.[12]

**EARTHY**, tasting: Ars., China, Ferr., Hepar, Ign., Merc., Phos., Puls.—2.

**EASY**: Acon., Ant. t., Arg., Aur., Cimic., *Coc. c.*, Colocn., Dign., Dulc., Euphr., Hepar, Iod., Kali b., Kalm.,[7] Kreos.,[7] Lach., Lact., Lip., Mag. m., Œna., Plumb., Puls., Ruta,[12] STANN., Staph., Sulph., Tellur., Verat.[12]—1 and 5.
— **day**: Ailan., Coc. c., Dig., Sil., Staph.
— **easier** after each cough: Aspar.
— **evening**: Dign.

**EASY**, **morning**: Arundo,[1] Mang.[7]
— on **motion**: Ipec.
— toward **night**: Melilotus.
— on **waking**: Melilotus.
**EGGS**, tasting like bad: Acon., Arn., Carbo v., Con.,[1] Eupion,[1] Hepar, Merc., Mezer., Mur. ac., Phos., Ph. ac., Sepia, Stann., Sulph.—5.
— tasting like yelk of: Kali c., Phos., Ph. ac., Sepia, Staph., Sulph.—2.
**EMPYREUMATIC** smell: Dros., Puls.—5.
— taste: Agnus, Bry., Nux v., Puls., Rhus, Squil., Sulph., Zinc.—5.
**EPITHELIUM**, exfoliated: Chin. s.
**EVENING** expectoration in the; morning without: Alum., Ant. cr., Arg., *Arn.*, Ars., Aur., Bar., *Bell.*, *Bov.*, Bry., Calc., Cann. s., Canth., *Caust.*, China, *Cina*, Coc. c.,[1] Digit., *Graph.*, Ign., Kali c., Kalm.,[1] Kreos., *Lyc.*, Mur. ac., Natr. c., Nitrum, Nux v., Œna.,[1] Rhod., Rhus, *Ruta*, Sepia, Sil., Stann., Staph., Sul. ac., Thuja, Verat.—5.
— in bed: Calc.
— before midnight on getting into bed: Sepia.
— **bloody**, when coughing: Natr. c.
— — after lying down: Sepia.
— — raising of blood, followed by copious mucus: Agnus c.[7]
— **grayish**: Lyc.
— **lumpy**: Kreos.
—, morning and night and evening with (daytime without) expectoration of yellow, green or gray, or of a milky-colored white, tenacious mucus, with, generally, a saltish, often a bitter, putrid, flat, sour or repulsive sweet taste and an unpleasant odor, which is swallowed; or, finally, of dark blood: Sepia.[2]

**EVENING**, **mucous**: All. c., Calc., Chin. s., Crot. t., Graph.,[6] Hydro. ac., Naja, Natr. m., Sulph.
— — 4 P. M.: Opun.
— — 5 P. M.: Caust., Hydras., Mag. c.
— **profuse**, on lying down: Graph.
— — heavy expectoration, evening on getting warm in bed: *Nux m.*[10]
— **taste**, bad: Lach.
— **scanty** and difficult sputa, which taste and smell like the secretions of a chronic catarrh; *Ign.*[2]
— scanty, yellow, jelly-like mucus, expectorated with difficulty, of a sweetish taste, sometimes with a little dark blood: Digit.[2]
— **tenacious**, yellow or bloody mucus (frequently copious): Iod.[2]
— **thick**: Kreos., Sulph.
— **white** mucus, which is tenacious and has a sour taste: Crot. t.[2]
— **white**: Calc., Crot. t.
— whitish, slimy, rarely somewhat bloody, almost tasteless substance, which is detached with difficulty: Cina.[2]
**FATTY** tasting: Caust., *Mag. m.*, Mur. ac., Puls., Sil.—2.
**FÆCES**, tasting like: Merc.[2]
**FAUCES**, comes from the: All. c., Aloe, Bry., Graph., Nux v., Sepia.
— — — forenoon: Lyc.
— — — morning: Lyc., Natr. m., Rei.
— — — while sneezing: Mezer.
**FETID**: Arn., Ars., Bell., *Calc.*, Carbo v., Cocc., Copaiva, Guai., Led., Lyc., Natr. c., Nitr. ac., Ph. ac., Puls., Sac. alb., *Sang.*, Sil., Stann., Sulph.—12.
— See also Offensive, Putrid, etc.
**FIRM**: Lyc., Mang., Wies.
— after breakfast: Ol. an.
— like leather: Kali c.
— in morning: Lyc.

**FISH**, tasting like: Acon.
**FLAKES** of: Ailan., Phos.
— discharge of purulent flocci, with erosive burning behind the sternum: Phos.[6]
**FLAT** tasting: Alum., Amm. m., Anac., *Ant. c.*, Ant. t., Arg., Arn., Ars., Aur., Bell., Bry., Calc., Caps., China, Copaiba,[1] Euphr., Ipec., Ign., Kali c., Kreos., Lyc., Natr. c., Natr. m., Par., Petro., Phos., Ph. ac., Puls., Rhus, Sabad.,[1] Sabin., Sepia, Stann., Staph., Stront., Sulph., Thuja.—5.
—, evening: Cann. s.
**FLESH**, tasting like putrid: Ars., Bell., Bry., Carbo v., Dulc., Kali c., Lach., Nitr. ac., Phos., Puls., Rhus.—2.
**FLIES** forcibly out of mouth: *Bad.*, Chelid.—2.
**FORENOON**: Bry.
— as from fauces, in: Lyc.
— **mucous**: Calc. s., Chin. s., Coc. c., Lyc., Sulph., Zinc.
— — 2 A. M.: Phos.
— — 3 A. M.: Opium.
— — 8–9 A. M.: Sil.
— — 8.30 A. M.: Spong.
— — 9 A. M.: Kobalt., Phyto.
— — 10 A. M.: Iris v.
— **purulent**: Œna.
**FREQUENT**: Agar., Asar., Chenop., Cina, Daph., *Euphr.*, Hepar, Iod., Lact., Laur., Lyc., *Puls.*, Ruta, Samb., *Senega*, *Sepia*, Sil., *Stann.*, Sulph., Verat.—12.
**FROTHY**: *Acon.*, All. c., Ant. t., Aqu. petr., Arn., ARS., Atropia, Canth., Chlo., Cot., Cubeba, Daph.,[12] Dios., Dros., *Ferr.*, Gad., Gastein, Hepar, Hura, Kobalt., Lach., Led., Linu., Millef., Natr. m., Nux v., Œna., Op., Pau. p., Petro., *Phos.*, Plumb., *Puls.*, Secale, Sil., Stram., Sulph., Thuja, Uran., Urt. ur.—1 and 5.
— morning: Sulph., Thuja.
— — 8 A. M.: Dios.
— — 9 A. M.: Cubeba.
— containing blood and mucus: Op.[7]
**GARLIC**, odor like: Ars.[2]
**GELATINOUS**: *Arg.*, Arn., Bar., China, Dig., Ferr., Laur.—4.
— See Viscid.
**GLAIRY**: *Arn.*, Carbn. h., Lin., Lip.
**GLOBULAR**: Agar., Calad., Coc. c., Selen., Sil.,[6] Thuja.—5.
**GLUTINOUS**: See *Viscid*.
**GRANULAR**: Agar., Calc.,[1] China, Hyper.,[1] Kali b., Lach., Lyc.,[1] Mang., Mezer.,[1] *Phos.*, Selen., Sepia, Spong., Thuja.—5.
—, morning, 3 to 4 A. M., or 4 to 11 P. M.: Lyc.
—, while sneezing: Mezer.
**GRAYISH**: AMBR., Anac., *Ars.*, Arum t., Cainca, *Calc.*, Carbo an., China, *Cina*,[4] Coc. c., Copaiba, Dig., Eupion, Gastein, Ham., Iod.,[7] Kali b., Kalm., *Kreos.*, Lac. ac.,[1] Lach., LYC., Mag. m., Mang., Merc. c., Natr. ars., Natr. m., *Nux v.*, Petro., Rhus, Seneg., *Sepia*, Sol. t. æ., Stann., Tabac., Tep., Thuja.—1 and 5.
— **evening**: Lyco.
— **green**: Copaiba, Rhus, Sepia.—5.
— — cold mucus, of putrid smell: Rhus.[7]
— — pieces: Sepia.[5]
— morning: Lyc., Natr. ars.
— **phlegm** as if mixed with dust: Phos.
— after **rising**: Phos.
— **yellow**: Anac., Lyc.—7.
— **white** mucus (seldom of yellow mucus), generally of a salty, sour taste: Ambra.[7]

**GREASY** taste: Alum., *Asaf.*, CAUST., Cham., Lyc., *Mag. m.*, Mang., Mur. ac., Petro., Phos., PULS., Rhus, *Sil.*, Thuja.—4.

**GREENISH**: Arn.,[7] *Ars.*, Asaf., Aur., Ben. ac.,[1] Bor., Bov., Bry.,[1] Cainca,[1] Calc., *Cann. s.*, *Carbo an.*, *Carbo v.*, Coc. c., Colch., Cop., Dig., Dros., *Ferr.*, Ham.,[1] Hyos.,[12] Iod., *Kali b.*, Kali c., KALI IOD., Kreos., Led., *Lyc.*, Mag. c.,[12] Mang., Merc., Merc. i. rub., Natr. c., Natr. m., Nitr. ac., Nux v., Œna.,[1] Ol. jec.,[1] Ox. ac.,[1] *Par.*, *Phos.*, Plumb., Psor.,[1] PULS., Raph.,[1] Rhus, *Sepia*, Sil., *Stann.*, *Sulph.*, Thuja,[7] Zinc.—5.

— grayish: Cop., Ham.,[7] Rhus, Sepia.

— — pieces: Sepia.

— morning: Ars., Crot. t., *Ferr.*,[6] *Lyc.*, Mang., Natr. m., Sil.

— — on awaking: Ferr.

— — when lying down: *Psor.*

— — 7 to 10 A. M.: Silicea.

— of a disagreeably sweetish taste, worse in evening: *Stann.*[6]

— tough expectoration, with cough and short breathing, from aching pains and palpitation: Cann. s.[6] (Clinical.)

— yellow: Kali b., Mang., Sil., Sulph.—5.

**GULPS**, by: Cupn. n.

**GUM-WATER**, like: Raph.

**HÆMORRHAGE**, hæmoptysis. See under Bloody Expectoration.

**HARD**: Con.,[2] Dig., Dign., Fago., Ox. ac.—1.

— after hawking: Sulph.

**HAWKED** up, mucus is: Agar., All. c., Amm. m., Ant. t., Bism., Calc., Caust., Carbo an., Chenop., Cina, Con., Croc., Crot. t., Dros., Ferr. m., Hepar, Iod., Kali b., Kali c., Lach., Lam., Laur., Lyc., *Marum*, Meph., Natr. m., Osm., *Par.*, Petr., *Phos.*, Ph. ac., *Plat.*, Plumb., Rhod., Rhus, Selen., Senega, *Sepia*, Stann., Tarax., Thuja.—12.

**HAWKING**, bloody expectoration on: Cham., Ferr., Hyper., Nitrum.

— constant gagging and hawking on account of viscid green mucus in larynx and trachea: Paris.[7]

— mucous expectoration after: EUPHR., Ol. an., Paris.

— gray salty expectoration from: Phos.

**HEAD**, from the: Dios.

**HEART**, anxiety in region of, amel. after copious expectoration: Ipec.

**HEAVY**: Kali b., Œna.

**HERBACEOUS** taste: Calad., Nux v., Ph. ac.—12.

— — and odor: Gels.

**HERRING**, taste like: Anac.

**HOUSE**, in the: Calc. s.

**IMPOSSIBLE**: Gels.

— see Difficult, Swallowed, etc.

**INDURATED**: Agar., Amm. m., Ant. cr., Bry., Calad., *Con.*, Iod., Kali b., Kali c., Kreos., Lach., Mang., Natr. c., Phos., Sepia, Sil., *Spong.*, Staph., Stront., Sulph., Thuja.—5.

**INFREQUENT**: Acon., Alum., Arn., Bell., Ign.—12.

**INK**, tasting like: Calc.[5]

**INODOROUS**: Arg.[5]

**INSIPID**: Alum., Amm. m., Ant. cr., Arg., Arn., Aur., Aur. s.,[1] Bell., *Bry.*, *Calc.*, Caps, *China*, Euphr., *Ign.*, Ipec., Kali c., Lyc., Natr. c., Natr. m., *Par.*, Petr., Phos., Ph. ac., Puls., Rhus, Sabina, Stann., *Staph.*, Stront., Sulph., Thuja.—4.

**IRON**, tasting like: Calc., Cupr., Sulph.[5]

**JELLY** like: Arg. Arn., Bar., Chin., Chin. s., *Dig.*, Ferr., *Laur.*—5.
—, copious and dotted with bloody points: *Laur.*
—, — in morning: Sulphur.
**LARYNX**, from: Aloe, Arn., *Carbo v.*, Ery. a., Natr. ars., Wild.—1.
**LEATHER**, odor like that of Russian: Arn.[2]
**LEMON** colored: Kali c., Phos., Puls.—5.
**LIVER** colored: Puls.[5]
**LIQUIDS**, expectoration from contact of liquids at back part of mouth: Amm. can.
**LOATHSOME**: Dros., Puls.—5.
**LIE DOWN**, profuse sputa of mucus, cannot lie down: Spong.[7]
**LOOSE**: Calc. ac., Carl., *Coc. c.*, *Lach.*, Merc. i. rub., Œna.
— day: Ferr.
— morning, after rising: Aralia.
— night: Gamb.
— Compare with Easy.
**LUMPY**: Acon., Agar., Ailan., Aloe, Arg. n., *Arn.*, *Ars.*, Bry., Calad., Calc. s., Chelid., Coc. c., Colch., Collin., Dig., *Hepar*, Lach.,[6] Kali b., Kali c., Kalin.,[7] Kreos.,[6] Lach., Lyc., Mang., Natr. m., Ox. ac.,[7] Par., Phos., Pimp., Selen.,[6] Sil., Sin. n, Sol. t. æ., Sulph.,[6] Thuja,[12] Verat., Wies. —1 and 5.
— copious expectoration of sweetish, frothy, white mucus with lumps in it: Kobalt.[7]
— after coughing: Osm.
— evening: Kreos.
— morning: Carb. ac., Lyc., Mang., Natr. ars.
— — 9 A. M.: Kobalt.
— tenacious (difficult and scanty), round lump, of cherry red color: Acon. (Pearson.)
— tough, round jelly-like lump, a yellow or soft brick shade: Bry.
**LUNGS**, from the: Aqua. petr., Aur., Chelid., Chin. s., Crot. h., *Phos.*
— deep from: Chelid.[12]
**MASSES**, in: Ars., Coc. c., Nitrum, Sin. n.
**MATTER**: Calc. ac., Gels., Kalm., Kali b., Natr. ar., Op., Psor.
— morning: Ferr. (v. Purulent).
**MEAL**, tasting like: Lach.[5]
**MEAT**, tasting like putrid: Ars., Bell., Bry., Carbo v., Dulc., Kali c., Lach., Nitr. ac., Phos., Puls., Rhus.—5.
— tasting like broth of: Iod.[2]
**MEMBRANEOUS**: Am. cau.,[1] *Brom.*, Hepar, *Kali b.*, *Merc. cor.*,[1] SPONG.—11.
**MENSES**, bloody expectoration during: Phos.
**METALLIC**, taste: Agnus, Calc., Cocc., *Cupr.*, *Ipec.*, Kali b., Kali c., Kreos., Natr. c., Nux v., *Rhus*, Zinc.—5.
**MILKY**: Amm. c., *Ars.*, Carbo v., Ferr., Plumb., Puls., Sepia, Sil., *Sulph.*, Zinc.—5.
— smell: Dros., Spong.—2.
— taste: Phos.
**MORNING**, in the: without expectoration in the *evening:* Acon., Alum., Ambr., *Amm. c.*, Amm. m., *Ang.*, *Ant. cr.*, Ant. t., Arn., Ars., Aur., Bar., Bell., BRY., *Calc.*, Caps., Carbo an., CARBO VEG., Caust., Cina, Colch., Cupr., *Dig.*, Dros., Euph., *Euphr.*, *Ferr.*, HEPAR, Hyos., Ign., Ipec., Kali c., Kreos., Lach., Led, Lyc., *Mag. c.*, Mag. m., *Mang*, Meph.,[7] Mezer., Mur. ac., Natr. c., *Natr. m.*, Nitrum, *Nitr. ac.*, Nux v., PAR., PHOS., *Ph. ac.*, PULS., Rheum, Rhod., *Rhus*, Seneg., SEPIA, Sil., Spong., SQUIL., Stann., Staph., Stront., Sulph., SUL. AC., Verat., Zinc., Zingiber.—1 and 4.

**MORNING, bloody** mucous expectoration in morning; bloody taste in mouth: Bell.[7]

— — blood-streaked, purulent or albumen-like, slimy, sometimes frothy or greenish expectoration of a sweetish-putrid or sourish taste: Ferr.[2]

— — light-red blood mixed with mucus, of a putrid-sweetish taste: Ipec.[2]

— — dark blood, or of tenacious whitish mucus of a sourish, herby taste—more rarely of an offensive pus: Ph. ac.[2]

— — dark blood, or of a thin, yellowish, sometimes blood-streaked mucus which generally tastes sour, but often also salt or sweetish: Sul. ac.[2]

— — at times mixed with blood, at times of blood alone; bitter, yellow, more mornings: Ailan.[7]

— — mixed mucus with blood, scanty, in morning after long coughing spell: Alum.[2]

— — mucus mixed with dark blood, scanty, of putrid taste and odor: Cupr.[2]

— — tenacious mucus, mixed with dark blood, and having a flat taste: Ant. cr.[2]

— — cough is excited by copious, flat tasting, watery mucus, sometimes blood-streaked, in chest and throat, which is difficult to dislodge, and can only be expectorated in the morning: Euphr.[2]

— **gray** green, in evening without, all other times with expectoration of acrid pus, or a gray-greenish cool mucus of a putrid, flat, metallic, sour or saltish taste; or else of bright-colored, lumpy, sometimes brownish blood: Rhus.[2]

**MORNING, gray,** yellow, or green pus, or of a milk-white, tenacious mucus, generally of saltish taste: Sepia.[7]

— **green** mucous sputa, difficult, worse mornings when awaking and evenings on lying down: Psor.[7]

— — greenish white casts, as if from air cells: Nitr. ac.[7]

— masses of mucus which are often purulent and bloody, and have generally a sour but sometimes a sweet taste, and, in the latter case, an offensive odor: Hepar.[2]

— muco-purulent; free in morning, sticky and scanty during day: Ailan.[7]

— profuse, purulent, offensive, green, tasting salty only in morning (expectoration lessens as patient gets worse): Sepia.[7]

— purulent expectoration of an offensive odor, and often of a bright-colored, frothy blood: Led.[2]

— purulent sputum and pain about last short ribs, left side: Natr. s.[7]

— **tenacious,** copious, mucous, albumen-like expectoration, of a somewhat offensive odor: Senega.[2]

— — scanty, tenacious, yellow or indurated mucus, of a scarcely perceptible sour taste, and which patient cannot eject but must swallow: Spong.[2]

— **yellow,** somewhat of a yellow, generally bitter expectoration which has to be swallowed: Dros.[2]

— — yellow, greenish, or purulent, sometimes brownish bloody expectoration, or, less frequently, a tenacious, whitish mucous or watery expectoration; the sputa have an offensive sour or saltish taste and unpleasant odor: Carbo v.[2]

**MORNING**, **yellow** mucus, often blood-streaked, generally with a flat, sometimes a sourish, more rarely a saltish taste: Natr. m.[2]

— — yellowish, tenacious, starch-like, often saltish mucus (less frequently *vice versa*): Bar.[2]

— — yellow or albumen-like, tenacious mucus, tasting like an old catarrh or somewhat saltish: Mezer.[2]

— — slight dislodgment of yellow or watery, crude mucus, of a fatty taste, which has to be swallowed; sometimes also of dark blood: Mur. ac.[2]

— — sputum of greenish or yellow lumps, easily raised in morning; or difficult, of tough mucus: Mang.[7]

— — very much whitish or reddish colored mucus, of a repulsive sweetish, sometimes empyreumatic taste and a somewhat offensive odor: Squil.[2]

**MORNING and day**, dark blood mixed with coagula, or of a yellow, acrid pus, of a bitter, sourish, or salt taste and offensive odor: Nitr. ac.[2]

— — — dark blood, or of yellow, greenish, purulent, often cold, or milk-white watery mucus, of, commonly, a sourish, or a putrid-flat or saltish taste, or like the offensive discharge of an old catarrh: Sulph.[2]

— — — dislodges with difficulty, scanty, watery, sometimes saltish mucus, which has to be swallowed: Lach.[2]

— — — mucus mixed with coagulated blood: Acon.[2]

— — — copious mucous or purulent, yellowish or grayish, sometimes bloody expectoration; generally of a sour taste and offensive odor: Calc.[2]

**MORNING and day**—(*Continued*).

— — — mucus, yellow or mixed with coagulated, brownish blood, often cold; has generally an unpleasant flat taste, and is at first difficult to dislodge: *Bry.*[2]

— — — purulent (lemon-yellow, gray, greenish or whitish) or bloody mucous expectoration, of a salt (also sometimes a bitter, flat, putrid, sweetish or sour) taste and offensive odor: Lyc.[2]

— — — dislodges tenacious mucus or yellow pus, of a flat, sweetish, or a sour taste, which must be swallowed: Kali c.[2]

— — — tenacious mucus copious, resembling the white of an egg, or else of greenish yellow pus, putrid, or sometimes a sour or salt taste, and an offensive odor: Stann.[2]

— — — tough whitish mucus, appearing as if mixed with dust, or of a yellow, pus-like or rust colored, often cold mucus, of a sour, salt or sweetish taste; or else of bright red frothy blood: Phos.[2]

— — — much yellow or greenish mucus of various taste (repulsive, bitter, empyreumatic, flat, putrid, fatty, salt, sour, like the secretion of an old catarrh, or like tobacco juice); often also of dark clotted blood: Puls.[2]

— — — yellow, purulent, blood-streaked, tenacious mucus, having a repulsive, sweetish putrid, or metallic taste, or of bright blood: Zinc.[2]

— — — copious yellow, somewhat greenish, purulent or tough, sometimes milk-white, acrid mu-

**MORNING and day**—(*Continued*). cus; more rarely of bright-red, frothy blood; generally of a fatty taste and offensive odor: Sil.[2]

— — — yellow, thin but tenacious mucus, or of dark blood, of somewhat saltish taste: Mag. c.[2]

—, **evening** and night with (daytime without) expectoration of yellow, green or gray pus, or of a milky-colored white, tenacious mucus, with, generally, a saltish, often a bitter, putrid, flat, sour or repulsive sweet taste and an unpleasant odor, which is swallowed; or, finally, of dark blood: Sepia.[2]

— after **rising**: Chin. s., Mag. m., Sepia, Sulph.

— on rising: Calc. s.

— after **waking**: *Sulph.*, Thuja.

**MOULDY**, taste and smell, in evening: Borax.

— taste: Led., Ph. ac.—2.

**MOUTHFULS** of mucus at a time, raises: Phos., Lyc.

— expectoration on contact of liquids with back of mouth: Amm. caus.

**MOVING**, copious expectoration when: *Ferr.*

**MUCOUS**: Acon., Æth., Æsc. h., Agar., Agnus, All. c., Aloe, Alum., *Ambr.*, Amm. c., Amm. m., Anac.,[2] Ang., Ant. cr., *Ant. t.*, *Arg.*, Arg. n, Arn., *Arum tr.*,[7] ARS., Atrop., Asar., Aur., Bad., Bar., Bell., Ben. ac., Bism., Bor., Bov., *Bry.*, *Cact.*, Calad., CALC., Calc. caus.,[6] Cann. s., Canth., Caps., Carb. ac., Carbn. s., Carbo an., Carbo v., Caust., Cham., Chelid., Chenop.,[12] CHINA, *Cina*, Coca, Cocc., Coc. c.,[12] Con.,[2] Cop., Croc., Crot. t., Cubeba, Cupr., Der., Dig., Dir., Dor., *Dros.*, *Dulc.*, Erio., Ery. a., Eugen.,[12] Euphr., Eupion, Ferr., Fran., Gastein, Graph., Guai., Ham., Hepar, Hyos., Hyper., Iber., Ign.,[2] Indigo, *Iod.*, Ipec., Kali b., Kali c., Kis., Kobalt., Kreos., Lac. ac., Lach., Lact.,[6] Laur., Linu., Lob. s., LYC., Mag. c., Mag. m., Merc., Merc. c., Merc. sol., Mezer., Mur. ac., Naja, Natr. c., Natr. m., Natr. sul., Nicc., Nitr. ac., Nitrum, Nux m., *Nux v.*, Œna., Olean., Ol. an., Ol. jec., Op., Osm., PAR., Petr., Phell.,[12] PHOS., Ph. ac., Physo., Pimp., Plan., Plumb., Puls., Raph., Ratan., Rheum, Rei., Rhod., Rhus, Rhus r.,[24] Ruta, Sabad., Sabina, Samb., Sang., Secale, Sed., Selen., *Senega*, *Sepia*, *Sil.*, Sin. n., Spig., Spong., *Squil.*, STANN., *Staph.*, Sulph., Sul. ac., Tarax., Tep., Tereb.,[7] Trif. p., *Thuja*, Upa., Ust., Verat., Wies., Xan., *Zinc.*—1 and 5.

— **acid**: Caust., Merc., Sil.—5.

— **air**, agg. in open: Kobalt.

— **afternoon**: Amm. m., *Bad.*

— — 4 P. M.: Opun.

— — 5 P. M.: Caust., Hydras., Mag. c.

— **albuminous**: Coc. c., Ferr., Mezer., Senega.—5.

— **bed**, when in: Amm. c.

— **bitter**: Ars.,[2] Cham, Puls., Sepia.—5.

— **black**: Kali b., Lyc., Ox. ac., Rhus.—5.

— **bloody**: Acon., Ailan.,[7] Alum., Amm. c., Anac., Ant. t., Arn., *Ars.*, Bell.,[16] Bism., *Bor.*, *Bry.*, Cact. gr., China, Cina, Collins., Con., Cupr., Daph.,[16] Digit., Dros., Dulc., Eugen., Euphr., Ferr., Hepar, Iod., Ipec., Kali c., Kobalt., Laur., Lach.,[7] Lachn., Lyc., Mag. m., Merc., *Natr. m.*,

**MUCOUS**, **bloody**—(*Continued*). Nux m., Op., Phos., Sabina, Secale, Selen., Sil., Spong., Squil., Sul. ac., Zinc.—5 and 12.
— — Compare with Blood-streaked.
— **bluish** lumps: Kali b.[12]
— **catarrh**, tasting like old: Sulphur.[5]
— **coating** lumps of coagulated blood: Collins.[5]
— **cold**: Bry., *Nux v.*, *Phos.*, Rhus, Sulph.—5.
— **copious**: Alum., Cact. gr., Caust, Chelid., Coc. c., *Dulc.*,[7] Hydras., Indigo,[6] Iod., Puls., Ruta, Seneg., Sepia, Squil.—5.
— — ropy, adhering to pharynx: Coc. c., Kali b., Lobel., Sticta.—2.
— — yellow: Cact.[5]
— **cough**, during: Natr. m., Nitr. ac., Osm., Phos., Sol. t. æ., Sulph.
— **day**: *Ars.*,[2] Calc. s., Mag. s., *Spig.*—1.
— **difficult**: Lach.,[1] Mang.[6]
— **dinner**, after: Alumen.
— **disagreeable** taste: Sepia.[5]
— **disgusting** smell: Copaiba, *Sang.*
— **eating**, after: Sil., Thuja.
— **evening**, in the: All. c., Calc., Chin. s., Crot. t., Hydro. ac., Naja, Natr. m., Sulph.
— **flat** tasting: Ant. t.,[2] Bry.
— **forenoon**: Calc. s., Chin. s., Coc c., Lyc., Sulph., Zinc.—1.
— — 4 P. M.: Opun.
— — 5 P. M.: Caust., Hydras., Mag. c.
— **frothy**: Ars., Ferr. Fluor. ac.,[7] Kobalt., Opium.—1.
— **globular**: Coc. c., Selen.
— — as large as a pea: Coc. c.
— — in globules, with blood: Selen.[12]
— **gray**: Ambr., Lach.,[6] Nux v., Rhus.

**MUCOUS**, **gray** green: Cop., Rhus.
— — white: *Ambra.*
— **green**: Carbo v.,[6] Ben. ac.,[7] Ferr., KALI IOD., Kali b., *Psor.*[7]—5.
— — gray: Cop., Rhus.
— — pale green, yellowish mucus in clots, almost without cough, early in morning: Mang.[6]
— — yellow: Mang.
— **hardened**: Ratan.,[6] Spong.[1]
— — thick, difficult to detach, in morning, from upper part of air-passages: Sul. iod.
— **hawking**, after: EUPHR., Ol. an., Par.
— **herbaceous**: Ph. ac.[5]
— **jelly-like**: Dig.[5]
— **lumpy**: Amm. m.,[6] Arg. n.,[6] Ars., Calad., Chelid.,[2] Indigo,[6] Kali b., Kobalt.,[7] Mang., Ox. ac., Selen.[6]—1.
— — with blood in centre: Collins.[5]
— **metallic** tasting: Zinc.[5]
— **milky**: Sepia, Sil., Sulph.—5.
— **morning**: Agar. m., *All. s.*, Alum., Amm. m., Ant. cr., *Ant. t.*, Aran. d., Aralia, Bell., Bor., Calc., Carbn. cl., Chelid.,[7] Cimex., Dig., Fluor. ac., Fran., Hipp., Ipec., Kali iod., Lyc., Mang., Ment. pi., Mur. ac., Natr. m., Ol. jec., Phos., Rei., Selen., Sil., Sulph., Tabac., Thuja, Vic.
— — 2 A. M.: Phos.
— — 3 A. M.: Opium.
— — 8-9 A. M.: Silicea.
— — 8.30 A. M.: Spongia.
— — 9 A. M.: Kobalt., Phyt.
— — 10 A. M.: Iris v.
— — bed, in: Ferr.
— — cough, during: Sulph.
— — getting up, after: Chin. s., Mag. m., Sulph.
— — waking, after: Sulph., Thuja.

**MUCOUS, mouthful**, raises, at a time: Phos., Lyc.
— **night**: Agar., Bell., Calc., Cycl., Hepar,[6] Sepia, Sil., Sulph.
— **purulent**: Ailan.,[7] Ars.,[2] Cop., Ferr., Hepar, Kobalt., Natr. c.,[6] Nitr. ac.,[7] Plumb.,[7] Staph., Zinc. —5.
— — See also *Yellow*.
— **putrid**: Arn., Ars.,[2] Cham., Ferr., Merc., Rhus, Stann., Sep., Sulph.
— — sweetish: Zinc.
— **reddish**: Coc. c., Kali b., Mang.,[7] Squilla.
— **ropy**: Coc. c., Ipec., Kali b., Lach.,[7] Lobelia.—5.
— **salty**: Alum., Ant. t., Ambr., Ars., Coloc.,[6] Hyos., Lach., Merc., Mezer., Phos., Samb.,[6] Sepia, Stann., Staph., Sulph.—5.
— while **shuddering**: Hyper.
— **sour**: Ambr., Ant. t.,[2] *Coc. c.*, Crot. t.,[6] Ferr., Hepar, Nux v., Phos., Ph. ac., Sulph., Sul. ac. —5.
— **starch**, like boiled: Arg., Cact., Dig.—5.
— **stringy**: Arum t., Coc. c., Hydras., *Kali b.*, Ruta.[1]—5.
— — and tough: KALI B.[6]
— **sweetish**: Calc.,[7] Ferr., Hepar, Laur.,[6] Nux v., Phos., Samb.,[6] Stann.—5.
— — sweetish flat tasting mucus: Anac.[7]
— — disagreeable: Sepia.[5]
— **tasteless**: Cina, Dulc.—5.
— **thick**: Ant. t., Calc., Coc. c., *Dulc.*, Kalm.,[7] Kreos., Op.,[6] Ox. ac., Phos., *Puls.*, Ruta, Sang.,[7] Senega, Sepia, Sil., Squil., Stann., Sulph., Thuja, Zinc.[5]—5 and 12.
— — yellow white, with central black lump of size of a pea: Ox. ac.[7]

**MUCOUS, tough**: Agnus, Ant. cr., *Ant. t.*, *Ars.*, Bell., Bry., Cann. s., Canth., Carbo v., Caust., Cham., Cistus,[12] Cocc., Coc. c., Euphr., Hepar, Iod., KALI B., Kali c., Kobalt.,[7] Mag. c., Mag. m.,[6] Mang.,[7] Mezer., Nux v., Par., Phos., Ph. ac., Puls., Ruta, Samb., *Senega*, Sepia, Sil., Spong., Squil., Stann.,[12] Staph., Verat., *Zinc.*—5.
— — and stringy: KALI B.[5]
— — thick mucus, mixed with blood: Kobalt.[7]
— **transparent**: Arg., Ars., China, Kreos., Laur., Phos.,[6] Sil.—5.
— when **walking**: Natr. m.
— **watery**: Euphr., Lach., Mag. c., Squil., Sul. ac.—5.
— **whitish**: Alum.,[7] Ambr., Apoc. c.,[7] Arg., Cina, Coc. c., Crot. t., Fluor. ac.,[7] Kali b.,[2] Kobalt., Kreos., Lyc.,[6] Nicc., Par., Phos., Ph. ac., Rhus, Senega, Sepia, Sil., Spong., Sulph.—5.
— white mucus attended by spasmodic asthma: Cupr.[6]
— — difficult to raise: Alum.[7]
— — streaked with blood: Borax.[7]
— **yellow**: Ambr., *Ang.*, Bism., Bry., Cact. gr., Coc. c., Copaiba, Kreos., Dig., Dros., Kali b.,[2] Mag. c., Merc., Mezer., Natr. m., Nux v., Ox. ac., Puls., *Ruta*, Spong., Staph., Sul. ac., Zinc. —5.
— — and copious: CACT. GR.
— — green: Mang.
— — — and stringy: Arum tr.
— — thick yellowish phlegm, with black lump in centre of size of a pea: Ox. ac.[6]
— — viscid yellowish mucus, forcibly ejected, flies out of mouth: Bad.[2]

**MUDDY-LIKE** pus, flies like batter: Phos.[12]

